# What is ethnographic about digital ethnography? A sociological perspective

**DOI:** 10.3389/fsoc.2023.1156776

**Published:** 2023-06-02

**Authors:** Peter Forberg, Kristen Schilt

**Affiliations:** Department of Sociology, University of Chicago, Chicago, IL, United States

**Keywords:** ethnography, anonymity, participant observation, digital ethnography, qualitative research, internet, hybrid ethnography

## Abstract

When COVID-19 health guidelines vastly restricted or shut down in-person ethnographic research in 2020, many researchers pivoted to forms of online qualitative research using platforms such as WeChat, Twitter, and Discord. This growing body of qualitative internet research in sociology is often encapsulated under the umbrella term “digital ethnography.” But the question of what makes digital qualitative research ethnographic remains open. In this article, we posit that digital ethnographic research necessitates a negotiation of the ethnographer's self-presentation and co-presence within the field that other forms of qualitative research, such as content or discourse analysis, do not require to satisfy their epistemological stance. To make our case, we provide a brief overview of digital research in sociology and related disciplines. Then, we draw upon our experiences conducting ethnographies in digital communities and in-person communities (what we call here, “analog ethnography”) to explore how decisions about self-presentation and co-presence facilitate or block the generation of meaningful ethnographic data. We think through pertinent questions such as: Does the lower barrier for anonymity online justify disguised research? Does anonymity generate thicker data? How should digital ethnographers participate in research environments? What are the possible repercussions of digital participation? We argue that digital and analog ethnographies share a common epistemology that is distinct from non-participatory forms of qualitative digital research—namely the need for the researcher to relationally gather data from the field site over an extended period of time.

## 1. Introduction

The proliferation of digital and interactive technologies such as social media in the 21st century has necessitated an expansion of the sociological toolkit for studying social life (Hampton, 2017). Platforms such as Twitter create opportunities for social scientists to gather data at a scale never-before-possible that can be used to assess questions such as how anti-immigration laws shape public sentiment (Flores, 2017), or to shed light on the drivers of political polarization (Bail, 2021). As the percentage of Americans who use social media grew from 5% in 2005 to 72% in 2021 (Auxier and Anderson, 2021), qualitative researchers have also adapted their research methods for a world in which there is increasingly less distinction between people's digital lives and their so-called real or in-person lives. While a few anthropologists delved into how people made community in immersive virtual worlds in the late 2000s (see Boellstorff, 2008; Nardi, 2010), researchers today face a cultural landscape where online lives and practices are increasingly normative and integrated into people's everyday lives (Bluteau, 2021). Illustrating such a shift, the late 2010s and early 2020s saw a rise in violent actions in the “real world” that resulted in part from misinformation shared at massive scales across digital platforms by proprietary social media algorithms and the use of the internet by extremists (Ndlela, 2020; Gaudette et al., 2022). To more fully understand how people's digital interactions co-exist with, shape, and are shaped by their lives offline, social scientists face the challenge of creating research techniques *for* digital technology rather than techniques conducted *through* digital technology (see Hine, 2015).

While anthropology, communications, media studies, and computational sociologists have been grappling with the opportunities and challenges of studying the interplay between virtual worlds, social media, and face-to-face interactions since the early 2010s (Boellstorff et al., 2012; Dicks, 2012; Hampton, 2017), qualitative sociologists entered these discussions with new vigor during the 2020 COVID-19 pandemic.^1^ As countries locked down shared in-person spaces, researchers began to follow people to “where the action is” (Goffman, 1969) on digital platforms. Without leaving home a sociologist could conduct rigorous and insightful qualitative research using Reddit forums, Instagram stories, and TikTok posts. A prolonged engagement with an online community could be accomplished at hours that fit the researcher's schedule, particularly for those with family and community responsibilities, and without the risk of viral exposure. The pace of interaction on a platform such as Discord could generate volumes of data in a week that might take 6 months to gather in a face-to-face setting. As the publishing expectations for sociology graduate students doubled in the last 25 years (Warren, 2019), digital ethnography also offered hope of faster and more efficient data collection for researchers worried about the slower tempo of in-person research.

The growing number of conference presentations, courses, and articles about digital ethnography in sociology suggests that the validity of examining mediated, networked life has become more widely accepted in the discipline (see Lane and Lingel, 2022). This growth in qualitative digital research is a much-needed corrective for the long-standing dismissal of online social life that has characterized mainstream sociology. At the same time, we question the use of “digital ethnography” as a catch-all term for any qualitative research done through social media platforms regardless of whether there was a participant observation component of the study. In thinking through what is ethnographic about digital ethnography,^2^ we offer the following proposition: generating meaningful data in a digital ethnographic study necessitates reflexive decisions about the researcher's participation and self-presentation that other forms of qualitative digital research do not require to satisfy their epistemological stance. In content and discourse analysis, for example, researchers convert existing social artifacts into data (Lune and Berg, 2012). In contrast, ethnographic research requires that “the observer is embedded in the data themselves,” a “reactive interaction” that means that “even the most passive researcher, merely by being present, inevitably shapes what is observed” (Small and Calarco, 2022, p. 12). It is the researcher's “co-presence” (Beaulieu, 2010)—the “exposure” to the social world and its people (Small and Calarco, 2022)—that makes a research project ethnographic, whether such prolonged, direct contact happens in physical spaces, through screens, or both. For digital ethnography, our increasingly “enmeshed” “digital landscape” (Bluteau, 2021) means that exposure to our participants and the digital technology in their lives can entail anything from becoming an orc in a virtual guild to posting “fit pics” on Instagram. We do not offer this proposition as a gatekeeping effort. Instead, we hope to offer a step toward greater epistemological clarity across qualitative digital methods—though we focus here on digital ethnography.

In this article, we use the term “digital” to reference forms of networked and mediated socialization.^3^ Digital ethnographic approaches could include participant observation in virtual or online communities, as well as interactions that bridge on- and offline worlds, such as hack-a-thons, esports tournaments, or workplace information systems. Of course, the ubiquity of social media platforms, proprietary algorithms, and artificial intelligence in our everyday lives can make it feel like all ethnography is now to some extent digital—or that it should be. Because technology is never evenly distributed across a population, however, we see value in considering digital ethnography as a distinct *mode* of ethnographic research that could be used alongside or in lieu of ethnography conducted solely in physical, in-person settings—what we will call “analog ethnography.” Sociologists today continue to produce distinctly analog ethnographies for a variety of reasons—digital interactions and records may be legally protected, such as in hospitals, less accessible to community members, such as among people who are unhoused, or purposefully restricted by structures of power, such as in prisons and jails. We do not make this distinction to position analog data collection as more authentic, as some audiophiles might say about classic vinyl records, or digital data collection as representing a brighter, more efficient future, as some technophiles might claim (Hassan, 2022). In contrast, we position analog and digital modes of ethnography as intimately related, increasingly overlapping, and intertwined while acknowledging that each mode has some unique affordances. While we will touch on “hybrid ethnography” (Przybylski, 2021), we marshal this ideal type comparison here to consider the possibilities, ethics, and outcomes of different researcher strategies across analog and digital ethnography.

In what follows, we draw on sociological writing about the craft of ethnography and our own fieldwork experiences to contribute to sociological conversations about “qualitative literacy” (Small and Calarco, 2022) and digital ethnography (Lane and Lingel, 2022). Forberg (2022) conducted a digital ethnography of QAnon, an anti-establishment conspiracy theory that began on the fringe web forum 4chan and migrated across the internet. Over the course of four months, Forberg used a new smartphone to follow online communities, tracking how his digital engagement influenced algorithmically generated app recommendations, newsfeeds, and notifications. He formed relationships with QAnon followers, conducted text and audio interviews, kept up with QAnon content and current events, and performed quantitative analysis on Twitter data scraped via an application programming interface. Schilt conducted an analog ethnography with people undergoing a major change to their embodiment, such as significant weight loss through surgical procedures. She negotiated access as a researcher to in-person conferences, instructional classes, and support groups designed to aid people in their life-altering transitions. Later, she conducted interviews with people undergoing these changes and their communities.

We start with a short overview of digital research in sociology. Then, we explore two major components that we see as central to an ethnographic epistemology: researcher self-presentation and ethnographic co-presence with community members. First, we consider the gains and trade-offs of being anonymous, pseudonymous, or known. Second, we consider what kind of data is generated by different degrees of participation, from being an anonymous “lurker” to a known participant. Of course, much ink has been spilled over the years about these considerations in sociological approaches to ethnography. We revisit these debates with an eye toward the experiences of ethnographers working in or across digital settings where anonymity, purposeful misrepresentation, and playful identity exploration is part and parcel of many community forums and where publicly identifying as a researcher can create a risk of being trolled or doxxed by disgruntled forum members at a speed and volume unprecedented in analog research.^4^ In our conclusion, we argue that a more “reflexive” (Abidin and de Seta, 2020) and intentional approach to ethnographic presence in the digital world can facilitate robust, ethical, and digitally-informed data collection that is applicable to settings across modalities.

## 2. Sociological approaches to digital life from Web 2.0 to Web 3

The study of social interactions is a central concern of qualitative sociological research. From Erving Goffman's “interactional ritual” (Goffman, 1967) to West and Zimmerman's “doing gender” (West and Zimmerman, 1987) to Randall Collins's “interaction ritual chains” (Collins, 2004), researchers have theorized how face-to-face interactions, both real and imagined, can create a shared sense of social reality, exacerbate stigma, bolster a sense of self, and generate collective emotions. Yet, as the internet became ubiquitous to the social life of many young people in the U.S. in the late 1990s—a cultural shift that DiNucci (1999) labeled as “Web 2.0”—sociological research and theorization did not keep pace. This emergent online culture was characterized by increased user-generated content, easy-to-create websites, and participatory engagement in new formats such as blogs, virtual worlds, massively multiplayer online role-playing games (MMORPGs), and nascent forms of social media. A few notable ethnographers recognized the importance of these new forms of sociality in the 2000s. In *Play Between Worlds* (Taylor, 2006), T.L. Taylor provided an in-depth look at the complex social networks of the community surrounding the networked multi-player game *EverQuest*. Taylor analyzed her own experiences playing the game with others online and her in-person participant observation at fan conferences. Boellstorff (2008) conducted a two-year ethnography within the virtual world of *Second Life* which, in 2007, had 8 million online inhabitants, and Nardi (2010) did ethnographic work in the *World of Warcraft* community, an MMORPG that had 10 million players in 2009. However, these hybrid and virtual approaches to ethnography did not become central to U.S. sociology for reasons we speculate have to do with the marginalization within the discipline of forms of engagement associated with the young, with games, and with play and leisure, as well as the dismissal of the interdisciplinary, progressive field of cultural studies in favor of the more politically neutral sociology of culture (see Long, 1997).

As usage of social media sites such as Facebook and YouTube spread across older age groups in the mid-2010s, quantitative sociologists began to take notice of these platforms as mechanisms for data collection (Hampton, 2017). The launch of Amazon's crowdsourcing website, Mechanical Turk, gave academic researchers a new format to reach millions of users willing to perform discrete online tasks. Rather than mailing expensive surveys or rounding up undergraduates for an experiment, sociologists could run their research projects with just a click of a button. Social media sites offered volumes of user-generated content. Computational sociologists sought data-sharing agreements with tech companies and used programs such as Python to “scrape” large quantities of data from user profiles and posts to learn about the formation of social networks, political views, activism, and consumer tastes. Data-scraping social media posts offered a way around the problems of both low response rates and social desirability bias, as researchers could access data made by users for public posting and metadata about users collected by companies. Rather than asking respondents about their social networks, researchers could pinpoint exactly how people were connected to one another via social media. The availability of these new data quickly raised thorny ethical issues about user confidentiality, proprietary algorithms, and online privacy (Parry, 2011).

Qualitative sociologists too began to explore how social media interactions factored into people's offline lives, such as Ilana Gerson's *The Break-Up 2.0: Disconnecting Over New Media* (Gerson, 2010). Studies examined how online content creators imagined their personas and their audiences (Marwick and Boyd, 2011), the offline repercussions of online interactions (Wang et al., 2011), and the expansion of virtual communities for marginalized and geographically isolated people (Gray, 2009). These studies mostly maintained the focus on people's lives offline, as the new affordances of technology still operated predominantly as an augmentation to face-to-face interactions. As social media usage and smartphones became ubiquitous in the U.S. in the mid-2010s, sociologists began to study how people's online interactions flowed into and shaped their lives offline—a more hybrid approach to ethnographic inquiry (Przybylski, 2021). Notable innovations came out of urban sociology, with researchers such as Lane (2019) and Stuart (2020) using analog and digital ethnography to trace how neighborhood violence in poor communities is driven by interactions on social media that turn deadly. Within studies of youth and schools, ethnographers such as Miller (forthcoming) and Outland (2020) explored how digital interactions inside and outside of school between students, parents, and teachers shape the conditions of students' learning environments in ways unimaginable only a decade ago. Such hybrid ethnography entails developing a long-term co-presence in a community's overlapping digital and analog settings, fulfilling Small and Calarco's (2022) expectation of ethnographic “exposure” to each space. In our view, these studies embody the type of research proposed by some of the early digital ethnographers because they abandon the arbitrary distinction between online life and “real” life that only “provide[s] *a priori* answers to some of the most intriguing questions” about the increasingly digital social world (Leander and McKim, 2003, p. 223).

### 2.1. What is digital about digital ethnography?

The emergent conversations about the shift to Web3 in the 2020s (Stackpole, 2022), a culture characterized by decentralization, immersive virtual and augmented reality (e.g., the metaverse and *PokemonGo*), block-chain technology, and artificial intelligence, have been accompanied by a formalization of digital ethnography as a method in its own right—an ethnography for the 21st century. Across the social sciences, researchers offer a host of digital ethnographic concepts to replace the analog language of field sites, such as “metafields,” “connection,” and “flows” (Leander and McKim, 2003; Hine, 2015; Airoldi, 2018). Yet, as with many emerging fields, we have seen a confusing proliferation of “buzzword ethnography” (Abidin and de Seta, 2020), wherein various forms or styles of ethnography are proposed for niche use cases. Buzzword ethnography—which includes methods such as “interface ethnography” (Ritter, 2021), “hashtag ethnography” (Bonilla and Rosa, 2015), and “appnography” (Cousineau et al., 2019)—has excelled at reimagining ethnography for specific, mediated environments. This platform specificity has a downside—namely a very short life span. Such fragmented theorizing privileges narrow digital use cases, some of which become antiquated with changing technology in less than 5 years.^5^ This approach, what we would call “media ethnography,” also often departs from the aim of analog ethnography to tell “thick,” “social stories” (Geertz, 1973; Murthy, 2008, p. 837), turning instead to digital stories where technologies rather than people become the subjects of ethnographic investigation.

We do agree with media ethnographers that a digital approach to ethnography should retain a sensitivity to the affordances of technology at the level of hardware and software, user interfaces, and entire platforms—essentially asking, how does digital technology work and what does that technology *do*? For us, a sociological approach to digital ethnography should relationally uncover how mediated social experiences engender behaviors, interactions, processes, and identities, connecting the distinctly digital with broader societal practices, norms, and experiences. Hine (2015) provides useful language for this linking of digital processes to social phenomena. She argues, extending the work of Geertz (1973) to the digital, that:

[T]he terms “experience-near” and “experience-distant” could usefully be rendered as “technology-specific” forms of engagement and “technology-neutral” forms of engagement. The ethnographer's task as a participant in a Facebook group is to bridge between the technology-specific status update, and the technology-neutral social act that the status update performs (p. 28).

Tufekci's (2017) research on social media activism in the mid-2010s is a good illustration of Hine's formulation. Much of the activist organizing that she studied took place on Facebook, a platform that requires first-time users to sign up with a verified name and encourages users to report violations of this rule to community moderators. Her research shows how activists who adopted pseudonyms for protection were targeted by opposition campaigns who spammed Facebook with reports as a method of censure. To apply Hine's (2015) concepts to this research, the *technology-specific* act of reporting on the platform enabled the *technology-neutral* act of political censorship. Tufekci extracts the social phenomenon from the technological process, which can help resolve the question of whether and how technology is creating new social patterns or reproducing and repackaging old ones (Seaver, 2017). Similarly, Phillips (2015) work demonstrates how the *technology-neutral* act of harassment is made *technology-specific* due to the anonymity afforded by the internet which enables the development of personas engaged in violent and hateful trolling. Finally, while Seaver's (2022) ethnography of music recommendation algorithms may be classified as hybrid because it took place in the analog corporate workplaces that create platforms such as Spotify and Pandora, he retained digital technology as his focal subject. When he shows that engineers create music recommendations by profiling users based on an assortment of data streams, he is describing the computational and industrial contexts that give rise to a *technology-specific* experience—one that forms the basis of a shared cultural experience for listeners worldwide.

What unites these ethnographies in our view is that the authors seek to “capture the complex imbrications of technology and society” (Sassen, 2002, p. 365) rather than to detail the function of a particular digital technology. Such studies investigate the affordances of digital technology by developing relationships to the people, places, and mechanisms of a digital community, and by participating in the technology at hand via these platforms. In doing so, the researchers capture something important about our increasingly networked world, leading some ethnographers to argue that “it is no longer imaginable to conduct ethnography without considering online spaces” (Hallett and Barber, 2014, p. 307), or to make a case that all ethnography is becoming hybrid (Przybylski, 2021). In contrast, we posit that there are still spaces where investigation does not require an attention to the social role of digital platforms, where such attention would not necessarily yield richer data, or where digital records are legally protected or restricted from sociological investigation. Hoang (2022), for instance, demonstrates the lengths that the very rich go through not to leave a virtual or physical paper trail of financial transactions when “playing in the gray.” Organizational and institutional ethnographies that center actors who handle legally protected or confidential records, such as hospitals, rely heavily on in-person modes of data collection. Some settings also actively restrict access to online life—we think, for instance, of Walker's (2022) ethnography of doing time in jail, which is the ultimate illustration of what it means to be trapped in “meatspace” with other people with no virtual escape. To imagine the social world that we currently inhabit as evenly hybrid runs the risk of excluding the experiences of people who are very rich, very poor, very old, or who are in institutional setting, which means we should think carefully about what mode or modes of ethnographic research are most salient to our particular setting.

### 2.2. Okay, but what is ethnographic about digital ethnography?

We envision a digital ethnography that blends technological analysis and sensitivity with the sociological epistemology of analog ethnographers—namely an emphasis on co-presence in the field and decisions about researcher self-presentation. Digital techniques such as content analysis, distant reading, algorithm auditing, or user experience research are useful tools in an ethnographer's toolkit but are not, we argue, ethnographic on their own. The crucial question for the researcher is what form of knowing the data collection strategy provides. Here, we juxtapose two studies to make our point: Panofsky and Donovan's (2019) digital discourse analysis of a white supremacist forum and Blee's (2003) analog participant observation of white supremacist groups. Panofsky and Donovan's research question centers on how avowed white supremacists made sense of new genetic testing technologies that typically reveal some amount of “non-white” ancestry. To answer this question, they went to the white supremacist website, Stormfront, after learning that users were challenging each other to prove their racial purity by publicly posting their test results. The affordances of anonymous digital spaces meant that Panofsky and Donovan could collect data from posts unobtrusively. They selected online conversations about test results at one point in time, which allowed them to see how community members bolster or challenge each other's identity management strategies. In contrast, Blee examined the social processes that lead women to enter, stay, and exit racial hate movements. Her initial historical research showed the crucial role white women had played in these male-dominated movements. Yet, the existing sociological research omitted any engaged discussion of women's roles. To fill this gap, her research question focused on how women's racist beliefs developed, how these beliefs shaped and were shaped by their family and romantic relationships, and whether they enacted their racist beliefs in their everyday lives. To answer these questions, Blee spent years reading materials from hate groups, attending in-person meetings and events, and conducting life history interviews.

Both studies take a sociological approach to understanding racist beliefs and attitudes. But the method of data collection they adopt shapes how they operationalize these concepts in fundamentally different ways. Blee (2003) incorporated textual analysis into her research about women in racial hate movements, as she used documents produced by these groups, such as fliers, pamphlets, and radio programs, to identify the public-facing discourse that members circulated. To see these discursive frames in action, she attended rallies and meetings. She built long-term connections with women leaders and members of these groups and was immersed in these worlds for more than two decades. Conducting life history interviews, she was able to learn about how women in the movement saw their racist beliefs develop and evolve over time and the different pathways in and out of the movement they took. Building in-person connections over time also gave her access to participants' affects—one of the affordances of analog ethnography that is harder to gain across screens or through avatars. She could consistently share space with the same people and observe their emotional displays and body language over time. Her inclusion of an ethnographic component of the study further allowed her to explore the possible contradictions between “saying” and “doing” racist ideologies, a distinctively ethnographic endeavor (Martin, 2003). Adopting this triangulated approach of textual analysis, life history interviews, and ethnographic participant observation positioned Blee to make an argument about how white women enter and exit hate movements, and how their ideological statements about race do or do not shape their everyday interactions.

Panofsky and Donovan's focus on online posts allowed them to capture how racist beliefs operate at the discursive level on the Stormfront website at one point in time. What they can see in these online conversations are how users collectively and publicly work through the cognitive dissonance produced by scientific information about racial ancestry (genetic tests) and racist ideological beliefs about the desirability of white racial purity. They can document the range of discursive strategies in a historical moment when far-right movements are simultaneously adopting and dismissing scientific understandings of race. They also can draw some inference about social hierarchy on the forum and the relative social standing of individual users by looking for patterns about which users receive support and which users receive ridicule after posting similar test results. What this study cannot do is to tell us anything about how these users came to their beliefs or whether they act on these beliefs in their offline lives. To get this kind of data, they would need to change methods. They could, for instance, approach users on Stormfront for interviews to learn more about their beliefs and attitudes. Digital researchers have gained access to criminal or far-right fringe groups with similar approaches (Gehl, 2016; Forberg, 2022). Or Panofsky and Donovan could build connections with users and begin to observe them in their offline lives. But—and this is crucial to our argument—*the absence of interviews or observations in a digital discourse analysis is not a limitation*. This choice reflects a particular approach to knowledge formation that we should not expect to mirror the epistemological stance of other forms of qualitative research (Lamont and Swidler, 2014; Small and Calarco, 2022).

Distinct digital research methods share some similarities, such as the unique affordances around researcher anonymity on many digital platforms, the access to large volumes of data, and ethical concerns about what is public and what is private online. Panofsky and Donovan's study, for instance, has a lot of overlap with Bail's (2021) research about far-right polarization on the internet, though the sample sizes in these projects are vastly different. But, we argue, analyzing digital content does not make a digital ethnography. We suggest that digital ethnographers share research opportunities, challenges, and ethical concerns with analog ethnographers that are not present—and do not need to be present—in other forms of digital research. Making this point, De Seta (2020) revisits Fine's (1993) classic “ten lies of ethnography” for the digital age to show how struggles with participant observation, insider knowledge, and the bounds of a field site shape ethnographic research in both modalities. While the large-scale content analysis and data-scraping developed during the rush for big data suggests that digital researchers can use the increasingly large and complex internet to “know it all,” digital ethnography is still grounded in small data that is collected relationally and dependent upon a negotiation between the ethnographer's engagement in the field and what the field can provide. Unlike content analysis's retroactive capacity to download and archive data (Angelone, 2019), the analysis gained through ethnographic engagement must acknowledge the researcher's dependence upon digital platforms, the opportunities and challenges of emerging and evolving relationships with anonymous users, and the researcher's own ability to be active in the field in the field over extended periods of time.

## 3. Sociological considerations for digital ethnography: self-presentation and co-presence

To make our case for a sociologically centered digital ethnography, we focus on two decisions that are central to an ethnographic research design: the degree of anonymity a researcher will have in their field site (self-presentation) and the degree of participation they will engage in over the course of their study (co-presence). As we show in our first section, digital ethnography opens new questions about the ethics of anonymity and disguise as a research strategy. Within the interdisciplinary field of digital research, these ethical issues have received much coverage, particularly around extreme cases where self-presentation strategies are closely linked with researcher safety, such as studies of criminal behavior on the darknet (Barratt and Maddox, 2016). In many cases, however, the ethics of creating a pseudonym for a web forum or reading a public thread as an anonymous user are left to Institutional Review Boards, who often know little about internet norms, and ethics guidelines offered by professional associations which may not have been updated for the digital age. We consider these ethical issues in light of the epistemological stance of ethnographic research in sociology. Thinking through the affordances of digital platforms alongside debates about the ethics of disguised research in analog settings, we explore the gains and trade-off of being anonymous, pseudonymous, or known in a field site.

Next, we examine the degree of participation a researcher adopts in digital and analog ethnographies. Co-presence provides the interactive, real-time perspective on events that makes ethnographic research unique from interviews or content analysis (Small and Calarco, 2022). However, decisions about how to participate are shaped by many factors, such as the feasibility of a particular approach in a setting, the positionality of the researcher vis-à-vis the community of study, and the degree of risk a certain strategy brings to the researcher and the respondents. Within digital ethnography, digital platforms also shape the possibility of participation and community norms for engagement in complex ways. What constitutes participation in digital ethnography—as well as when active participation is appropriate—are integral and unresolved questions for contemporary sociological research. We conclude with a discussion of the unique ethical quandaries faced by digital ethnographers when considering the potential risks of online participation to themselves, their respondents, and society.

### 3.1. On the internet nobody knows you are a sociologist: digital anonymity

As one of the possible tools in a researcher's methodological toolkit, ethnographic observations present a way around a research quandary that has long kept social scientists up at night: the gap between what people say they do in interviews and on surveys and how they behave consciously or unconsciously in their everyday lives. Within a workplace or a school, for instance, people might tell an interviewer that they deeply believe in gender equality or meritocracy. Observing these same people over time, however, an ethnographer may see them engaging in behaviors that reproduce forms of stratification (Martin, 2003; Khan and Jerolmack, 2013). This extended engagement in a setting captures interactional mechanisms in a unique way, as the ethnographer functions as the research instrument (Small and Calarco, 2022). At the same time, openly observing people can have a chilling effect on behavior. Researchers often adopt some degree of a cover story to get meaningful data. The depth of a cover ranges from “shallow,” an approach where the researcher is known to be collecting data, but the exact questions of interest are slightly obscured, to “deep,” where the researcher is a total participant in a setting where most people do not know that they are being observed (Fine, 1993). Studying the Levittown suburbs, Gans (1967) adopted a shallow cover by telling neighborhood residents he was conducting a historical study. Seeking to observe customer interactions in a big box toy store, in contrast, Williams (2006) adopted a deep cover where her co-workers knew her as just another cashier. As ethnographic relationships are always evolving, a researcher may move between no cover, shallow cover, and deep cover over the course of a single research project (Hoang, 2015).

While deep cover, a form of disguised research, is not explicitly forbidden in sociology, this approach is governed by special considerations from the American Sociological Association (1999). Disguised researchers must anonymize research subjects and settings and consider a specific set of questions about whether this approach will increase possible harm to respondents during and after publication. As long-standing debates in sociology demonstrate, however, there is not a definitive answer to the question of what constitutes an ethical disguise. We are accustomed to researchers making strategic choices about their presentation of self in face-to-face research, such as wearing more professional clothing than they might in their everyday lives or displaying buttons or clothing styles that locate them within a shared subculture with respondents. But what if a researcher goes along with sexist conversation that he does not agree with to build rapport with law enforcement (Leo, 1995)? Is that an ethical cover? Taking a more extreme case, sociologists would widely agree today (as would the Institutional Review Board) that it is unethical to ask graduate research assistants to join a doomsday cult as undercover participants (Festinger et al., 1956). At the same time, some disguised studies that raised hackles in sociology generated insights into inequality and discrimination that would not have been possible through other methods at that time (Nardi, 1996). Yet, with the emerging conversations about unmasking field sites in sociology (Jerolmack and Murphy, 2019) and the push for greater data transparency (Murphy et al., 2021), analog ethnographers who adopt a deep cover face increased scrutiny from reviewers, IRBs, and the public.

Expectations about identity and self-presentation on the internet present new opportunities for deep cover. Beginning with the earliest “cyber” ethnographies, researchers have explored how people experiment with identity online (Turkle, 1995; Kendall, 1998). Identity experimentation can mean developing anonymous personas distinct from one's “real self,” a topic widely explored in research on online political harassment and trolling (Phillips, 2015; Bail, 2021). It can also mean developing an online persona that is a bridge to a future self outside of these networked spaces, as is the case for some in the non-binary and trans community (Brown, 2019). Anonymity remains the default experience on many modern digital platforms, such as MMORPGs, the darknet, and forums including Reddit and 4chan (Phillips, 2015; Barratt and Maddox, 2016). These situational norms lower the barriers for disguised research online. While the graduate student researchers of the 1950s had to remember the details of their cover story in daily face-to-face interactions with members of the doomsday cult, the research assistant of the 2020s can create an online persona, sign up for a public forum, and begin integrating herself into one of many doomsday prepper groups as a novice. She could watch conversations unfold online and take screenshots of interactions as a record, rather than hiding in the bathroom of the cult leader's home frantically taking notes. Anonymous or less traceable platforms also can provide a researcher with greater access to hard-to-reach or criminal groups and may offer more protection to the researcher from being doxxed by community members (Gehl, 2016). Of course, this norm of anonymity also presents challenges to researchers who are unable to determine the demographics of the people whose hashtags and posts they are analyzing. Researchers can draw some inferences about, say, the gender of participants on an incel chat room based on avatars and shared details, but it is hard to verify these assumptions. This lack of identity verification for posts and online interviews can be a barrier to publishing digital research in sociology, as reviewers typically expect demographic information even for “small n” studies that do not purport to offer generalizable conclusions.

Community norms that privilege digital anonymity mean that users are less likely to expect that someone posting on a forum is who they say they are—as captured in the long-standing meme that nobody on the internet knows you are a dog—or to assume that a username like @sexyboi47 reflects anything about the user's physical presentation (or that the user is even a human). These digital norms may ease a researcher's concerns about the ethics of entering these spaces without acknowledging himself explicitly as a sociologist. Some researchers go so far as to argue that such an acknowledgment in a digital setting that privileges anonymity violates community norms and puts the researcher and the community at risk (Ferguson, 2017). While this may be true in some cases, our point here is to consider whether using this strategy generates more meaningful data than being known as a sociologist in an online space. We work through this question using Forberg's digital ethnography of QAnon, a political conspiracy theory primarily associated with the far-right which alleges that an anonymous user on the fringe internet forums 4chan and 8kun has insider access to former U.S. president Donald Trump's plans to overthrow a Satanic cabal controlling the world. QAnon followers believe that fervent internet activism will usher in a political and religious Great Awakening, which has resulted in a proliferation of QAnon communities across social media platforms (Forberg, 2022). While followers are eager for new recruits, however, they are often paranoid about outside infiltration by researchers, the mainstream media, and government agencies.

Forberg began his study by accessing the community through anonymous accounts that he created for research purposes. This decision reflected the community norms he observed others using in Q forums. As a researcher, he was disguised in the sense that Q followers did not explicitly know that his accounts were tracking their posts and conversations for research purposes. He followed users on various social media platforms to cultivate a trackable internet presence that would produce algorithmic recommendations and experiences potentially similar to those of QAnon followers. This method provided a great deal of insight into the public structure of the QAnon movement, as it allowed Forberg to see how conspiratorial discourses developed, what kind of content and online personas gained traction, and how the network of QAnon influencers attempted to break into the mainstream. This attention to different platforms also gave him a deeper understanding of the digital processes that QAnon members relied upon to spread their propaganda. To maintain his own ethical standards, Forberg did not use his anonymous accounts to gain access to aspects of the community that were not publicly accessible. If he wanted to message followers or join private groups, he used an identifiable account that could be traced to his researcher profile. After a few weeks he abandoned anonymity all together. While identifying himself as a sociologist did result in the occasional “block” from a Q follower or a nasty direct message, he found that this strategy overall did not prohibit him from access to the community, a point we discuss further in the second section.

In contrast, Schilt did not consider adopting an anonymous or disguised strategy in her analog ethnography of major life changes because this strategy felt unequivocally unethical in this setting. When she began researching the experience of undergoing a major life change, such as significant weight loss, she recognized that she was an outsider to the experience. While her main project was interview-based, she hoped to use ethnographic observations to learn more about the range of experiences people had and the language they used to talk about their own lives before she began interviews. She sought out free support groups, a forum where she imagined she might be able to observe a wide range of experiences from people across social class lines—a similar strategy to Forberg's initial observation of digital Q forums. Her preliminary research revealed, however, that while there were many free groups, the groups were not open to the public in the sense that just anyone could drop in. Most of the groups were run by a therapist or counselor who decided whether someone fit in the group based on their explicitly disclosed personal experience. This gatekeeping meant that there was no way to come as an anonymous participant. She did look at online forums but found similar expectations around participation.

Schilt had two choices at this stage. She could identify herself as a sociologist and ask to attend the groups as a researcher, a strategy she guessed would yield access to at least one group. The second strategy was to create a cover story related to one or all of the cases she was researching and seek access to the group in disguise. This approach is not without precedent in sociology (Lofland and Lejeune, 1960). But Schilt deemed this second strategy unethical, as we imagine most sociologists today would. Her goal was to attend these groups over time, not as a one-off event. As the norms of support groups typically include encouraging everyone to share, she likely could not stave off contributing a fake experience to a therapeutic setting for more than a few weeks. Contributing a fake experience in a setting where people shared deeply personal experience could generate a false sense of connection—which might be good for research but was bad for Schilt's ethical sense of self. She could have unwittingly provided erroneous information about a medical procedure or created anxiety by sharing a story that was too negative or too positive. Even if no immediate harm would come to support group members during the meeting, they could feel a deep sense of trust violation after the publication of a book or article. Schilt decided to adopt the first strategy of asking for access as a researcher. Most of the time, the group leader decided not to admit her as an observer. But she did get access to a few groups, which we discuss in the next section.

We do not offer these two examples to make a case for or against anonymous research, disguised research, or deep cover. There are many examples in sociology of ethical, disguised research in workplace ethnographies. However, these ethnographies are a far cry from a researcher pretending to adhere to the beliefs of a cult and moving in with them—a strategy that is likely to bring emotional duress to the researcher regardless of the quality of the data. Our point is that there are context-specific expectations about anonymity and privacy that should inform the balance between gathering the richest ethnographic data possible and doing the least harm to the researcher and the respondents. In Forberg's work, user anonymity was the assumed pretext for engagement on Twitter while Facebook users expected names and profile pictures to correlate to a real person. In Schilt's work, support groups members could be anonymous in the sense that they only used first names in the group. But the expectation in these settings was that people shared a personal experience, such as weight loss surgery, in common. This assumption that people are who they say they are shapes most interactions in face-to-face settings, even though the amount of personal information people have about each other might be minimal (Garfinkel, 1967). In contrast, the default assumption that people are good actors with a shared sense of reality in face-to-face interactions does not govern most digital spaces. And those assumptions matter when considering the ethics of anonymous and disguised ethnographic research.

At the same time, anonymous or disguised research that carries low ethical risk cannot be assumed to generate richer data than no cover or a shallow cover. If we set aside the ethics of Schilt's case, adopting a disguised strategy would have generated *more* data as she likely would have had access to a wider range of support groups. But it is an open question as to whether she would have gotten thicker data. While she can never know what she was unable to access by being known as a researcher, she still gained a nuanced understanding of the ways that people talked about their experiences of major life changes across several settings—the goal of her ethnographic project. In Forberg's case, the expectation of online anonymity in his setting created ready opportunities for preliminary reconnaissance of the QAnon community. But this strategy did not generate deep ethnographic inquiry over time. On Twitter, where bots were common and sometimes indistinguishable from highly active, real QAnon followers, this technique mainly gathered public stories that lacked nuance. Without engaging community members, accessing private communities, or breaking the public veil of the community's activity, Forberg's initial research was akin to a content analysis of a living archive. Forberg used this tactic to learn more about the way that digital platforms functioned within the QAnon movement than about participants' experiences of the conspiracy theory—indeed, interviewing participants often demonstrated how misleading internet users' online personas can be (see Bail, 2021). If we were to set ethics aside once again, Forberg would have likely gathered *different* data by faking his way into private QAnon spaces—especially those run by viral promoters or white supremacists—but the salacious nature of this data may not have been any thicker or more useful to his research questions. Even as a self-identified researcher, he found that the more extreme QAnon followers he interviewed were comfortable being candid about their fringe beliefs and practices, and about their negative interpretations of him.

The fact that many digital spaces enable greater flexibility in self-presentation than analog spaces does not mean that ethnographers should always be taking advantage of this flexibility. The appeal of digital disguises is evident: In describing researchers' *ethnographic toolkit*, Reyes (2020) highlights how ethnographers' “visible (e.g., race/ethnicity) and invisible tools (e.g., social capital)” (p. 221) can be *strategically* employed to relationally access field sites and build rapport with participants—opening, closing, and keeping open doors.^6^ Anonymity is a viable strategic decision in both analog and digital spaces, as Schilt could attend conferences and Forberg could browse web forums without making their identities known. But a researcher who attempts to enter a physical space for which they do not have the expected “visible tools,” such as a woman seeking access to a bathhouse that caters to gay men, will likely face barriers. Applying Reyes' toolkit to digital ethnography, however, it would be easier to overcome these barriers by *strategically* creating profiles that establish the researcher as an insider. Yet, this strategy too creates ethical dilemmas for the ethnographer, who we expect to begin developing relationships, building rapport, and conducting interviews—but now through this insider caricature. While we believe that there is room for playing with identity in digital ethnography, especially since this already strategically occurs in analog ethnography (Reyes, 2020), such a strategy is not necessary for gaining quality data. For us, once an ethnographer uses a disguised persona to engage with participants, he betrays the “principle of care” (Boellstorff et al., 2012) owed to participants and increases the risk of doing harm to the community he studies and himself. We turn now to these thorny issues of participant engagement in analog and digital ethnography.

### 3.2. Smash that like button! Liking and lurking in digital ethnography

The feature of ethnographic research design that distinguishes it from other qualitative methods is some degree of researcher participation in the particular social world or field of interest (Emerson et al., 2011; Small and Calarco, 2022). The role of the ethnographer, which can range from a peripheral observer to a complete participant, structures interactions in the field in ways that shape the type of questions she can ask and the data she is able to collect (Adler and Adler, 1987; Fine, 1993). Some ethnographers have made a case for embodied “carnal” participation in a field site—a “sociology of flesh and blood” (Wacquant, 2015, p. 1). Rather than observing how people train as boxers, for example, a researcher can use his body as a research instrument by training as a boxer alongside other community members (Wacquant, 2015). Embodied ethnography offers the promise of a closer approximation of habitus development in the Bourdieusian sense. As the ethnographer-turned-dancer sees his body and instincts change through training, for instance, he learns something about unconscious embodied practice that can be difficult to get through observations alone or interviews (Hancock, 2013). This approach is infused with assumptions about ability and access, however, that are rarely acknowledged. There is also a gendered and racialized component to the reception of carnal sociology. White people (mostly men) engaging in embodied ethnographies in urban settings receive an ethnographic premium in which they are lauded by the discipline for getting their hands dirty, so to speak, with deviant or criminal subcultures (Chancer, 1993; Small, 2015). In contrast, women engaging in embodied participation in a sexualized field site, such as a strip club or a hostess bar, face prurient questions about how far they went to get good data (Frank, 2002; Hoang, 2015). The pressure to prove oneself as an ethnographer through intensive in-person participation can compound the sexual harassment and violence that minoritarian ethnographers face in their research settings (Hanson and Richards, 2019).

Embodied participation can enable the researcher to become the phenomenon of study to some extent—though how close that approximation comes is an open question. The researcher is always a tourist in the sense that he returns to his home to write notes and can exit the world of study. He may be marked by his engaged, embodied participation with, say, new skills or new tattoos, but his simultaneous doing and observing separate him from the people he studies even if he shares their positionality to some degree. There also are ethical limits to what we can know as ethnographers and how we might go about this knowing. These ethical limits are, perhaps, more clearly delineated in analog ethnography because of the length and depth of these conversations in the discipline. To this point, we work briefly here through an example from Schilt's analog study of how people experience significant weight loss after a surgical procedure. Schilt could not become the phenomenon in this case because she did not qualify for weight loss surgery. She also did not attempt to approximate the experience of being heavy as some ethnomethodologists have done to learn more about the experience of being differently abled (Goode, 1994). Recognizing both the ethical quandaries and physical impracticality of an embodied form of participation, Schilt elected to attend support groups for people who had undergone weight loss surgery. She found that the experience of significant weight loss in a relatively short period of time made many group members “practical methodologists” (Garfinkel, 1967) in that they thought deeply about how this weight loss transformed their habits—how they ate, how they exercised, how they dressed—and their habitus—the way they inhabited their bodies and how they navigated physical space.

As the concept of a sociology of flesh and blood is distinctly analog, a prioritization of the carnal may seem to leave digital ethnographers out in the cold. From the cyberpunk world of Gibson's (1984) *Neuromancer* to Mark Zuckerberg's utopian vision of the 2020s metaverse, the allure of virtual worlds for many people is the ability to leave the body and its infelicities behind. But the digital ethnographer is still embodied, whether working on a laptop or participating in virtual interactions. Even the most basic forms of participation in online communities, which are text- or emoji-based, “liking,” retweeting, or upvoting a post, comment, or video, are embodied interactions. Digital platforms use these interactions to determine the spread of content across their site and to promote similar content to their viewers. YouTubers or Instagram influencers who have a monetized channel or page encourage this sort of participation from viewers, often ending videos with a loud, “Don't forget to smash that like button!” to generate more subscribers. A digital ethnographer can use these forms of online participation as a way into a setting, to build connections with community members, or to study the recommendation algorithms generated by user engagement (Forberg, 2022). Researching speed-running communities organized around the video game *Super Mario Bros*, for example, a researcher could watch videos of runs on YouTube. He might subscribe to the channel of a speed-runner and comment on videos as a way into the community, or he could like speed-running videos to see what else gets recommended to him from the YouTube algorithm. In this case, the ethnographer is still making decisions about his degree of participation in a setting based on the local norms and practices, but how he is able to enact that participation is shaped by the particular digital platforms he is working through. And none of these forms of participation get at the habitus of the speed-runner, which may require the incorporation of an autoethnographic embodied approach as seen in early sociological studies of videogame play (see Sudnow, 1979).

Anonymity norms in online spaces also facilitate a form of unintrusive participation that can be difficult to achieve in analog settings: lurking, the act of creating a profile on an internet forum or social media site but rarely engaging with others (Nonnecke and Preece, 2003). Lurkers may want to learn more about a medical condition, for instance, or like to follow the drama of the Twittersphere without fear of drawing ire over an unwitting comment. Embedded within communities of hundreds or thousands of users and followers on an account or on a platform, online lurkers may attract notice only from market researchers curious about how to monetize their attention. In contrast, analog lurking, which we imagine as observing interactions in a setting without engaging anyone, can raise immediate concerns from the people being observed. Sending students to take field notes in a grocery store or on public transit has long been a training exercise in sociological methods courses. But, with the constant refrain of “if you see something, say something” echoing across urban spaces in the U.S., this exercise can be rife with potentially dangerous misunderstandings. People may assume that a woman observing a group of children at the park is a mother or babysitter but immediately challenge the legitimacy of a man in the same space. Ethnographers of color, disabled ethnographers, and trans or non-binary ethnographers can face scrutiny and harassment from customers and security guards during a public observation exercise that white, able-bodied, cisgender researchers are less likely to face. This is not to say that analog ethnographers do not adopt degrees of lurking in their research. Such a tactic may be used in settings where voyeurism is a legitimate community role, such as in bathhouses catering to gay men (Tewksbury, 2002), or where many people are simultaneously observing an action or event, such as a protest (Tufekci, 2017). But the feasibility of this approach is always shaped by the formal and informal social rules of the space and the positionality of the researcher vis-à-vis other people in the setting.

The boundaries between lurking and peripheral participation are fuzzy in digital ethnography, where a researcher can upvote a Reddit post or follow a Twitter user without making a textual contribution to a setting. While it may be easy to identify active participatory acts—such as direct messaging forum members or commenting on YouTube videos—the boundaries between lurking and peripheral participation online require sensitivity to social context and an awareness of the specific functions of a given digital platform. Anonymously browsing the static, archivable forums typical of the early internet may feel hardly participatory, while the responsive, ephemeral nature of algorithmically-driven platforms such as TikTok makes any time spent online a fleeting ethnographic opportunity—yielding relational data that is dependent upon the ethnographer's presence and engagement with socially-networked systems. In Forberg's QAnon research, he transitioned from anonymous lurking to known participation over a short period of time. While he initially adopted an anonymous username, his presence in multiple Q forums became a source of suspicion and confusion among already suspicious participants. Forberg had started to direct message some participants to request interviews, at which point he disclosed his name and his university affiliation. As information traveled quickly in these forums, he made the decision to put his name on his profile for consistency. Prior to making this change, he did his own research into what information was publicly available about him in case he was doxxed by a community member and prepared himself for a backlash. Some people did look him up and post information they found about him on the forum, namely that his thesis adviser was a gender theorist (gasp) and that his research had been funded by an organization that sounded Jewish to the poster. A few times he received angry tirades in his direct messages about his presence as a researcher. More frequently, he was blocked by people he reached out to, likely due to his association with what Q followers saw as a liberal academic institution.

While abandoning his anonymous persona opened up Forberg to angry messages and limited his access in some ways, he felt that it made for more genuine engagement with potential interview participants who could ask questions about his background and work. Further, the few tirades he did receive from angry Q followers provided new insight into the social performances of QAnon—specifically how followers defended themselves from perceived threats and upheld their belief in Q as indicative of moral and intellectual superiority. Over time even some of the initially angry respondents agreed to an interview due to an appreciation for his curiosity and honesty. At the same time, Forberg's experience with this approach is inseparable from his positionality vis-à-vis the field. QAnon is a predominantly white, cisgender community, and members likely read Forberg to be “like them” even if he was attending an elite university. His visible status as a white man on his profile pictures allowed Q followers to project their own values onto him. Some assumed he was an ideological ally and joked at the expense of academics and progressives, while others recognized him as a distinct outsider and found it interesting to compare their beliefs to his own. This plasticity of Forberg's identity via participants' interpretations of him granted access to the QAnon community that another researcher with different social characteristics may not have gotten. People in minoritarian communities face far more online hatred and threatened violence than white, cisgender men (Vogels, 2021). We use Forberg's research, along with other studies of internet trolls (Phillips, 2015) and darknet users (Barratt and Maddox, 2016), to demonstrate that being known as a researcher in a fringe digital space can generate rich, interactional data that we see in many analog ethnographies. But this strategy comes with the risks associated with having a presence of any kind on the internet and the possible risk to the ethnographer from online abuse should be considered at the start of the study and continually re-assessed over time.

Schilt's role in support groups also ranged from peripheral participation as a known researcher to anonymous lurker but carried less personal risk. Unlike Forberg's case, Schilt's connection to an elite university lent her credibility, even when she was not given access to a group. When she did attend a group, the group leader would introduce her as a sociology professor in her first meeting or ask her to introduce herself. During meetings, she adhered as much as possible to the norms of the setting. If she observed people taking notes, she took notes. If no one took notes, she made minimal jots and then recorded voice memos in her car after the meeting. As a white, cisgender woman in multiracial and mixed gender spaces, she did not draw much attention with her presence. She nodded when people spoke, laughed at jokes, and smiled, all forms of common peripheral participation in support groups. To a newcomer who missed her initial introduction, she could come off as just another participant who never spoke (an analog lurker). When possible, Schilt introduced herself to newcomers to make her role clear. And if someone asked her a question that assumed she had a shared experience of weight loss, she quickly explained her presence as an outsider. Over her time in the group, regular attendees began to engage with her more during the free time before and after the meeting, referencing events from previous sessions she had witnessed or making jokes with her. Once when Schilt missed a meeting, a member of the group contacted her via email to tell her a funny story about something that happened that night, demonstrating that her presence had become expected. Schilt felt that her peripheral participation allowed her to make connections with people, and to gain an in-depth understanding of how significant weight loss shifted people's sense of self and social interactions. People knew who she was, so they could have sent her negative emails or text messages if they wanted to. She did not experience this, however, likely due to the group leader vouching for her at the onset and her lack of verbal participation.

We end this section by thinking through the ethics of participation in digital and analog ethnographies. While analog ethnographers may push for a sociology of flesh and blood, it is widely acknowledged that some forms of embodied participation, particularly disguised participation, are unethical. Returning to Festinger et al.'s (1956) study of a doomsday cult, it is hard to imagine a research design today that would send graduate students to participate as cult members—particularly when that participation took the form of pressuring new recruits to give up their worldly possessions and their children in preparation for the end of the world. While you as the researcher could be fairly certain the world was not going to end and that your assistants could leave the cult after doomsday failed to happen, the stress you caused your assistants and the harm they may have caused to cult members would not outweigh the empirical findings of your study. A known researcher engaging in embodied ethnography can bring harm to community members, such as sexual involvement with respondents that results in an unintended pregnancy (Goode, 2002), or to themselves, such as getting a broken nose in a sparring session at the boxing gym (Wacquant, 2015). Our point is that we have developed more of an ethical barometer for what we should and shouldn't do in analog ethnographic participation than we have developed for digital ethnography.

Such an ethical barometer for digital ethnography is difficult to assess. Some of the major questions of digital social science research are about the extent to which digital actions can cause “real world” damage. The unique affordances of the digital environment means that even peripheral participation or just a few likes here and there can cause societal harm at a scale unimaginable in analog research. Liking a post, as @Peterforberg or @sexyboi47, pushes up this content online, making it more accessible to others in their feeds. If you are studying K-Pop stars, reposting a popular video or liking the account of a singer may be an innocuous drop in the ocean among the digital engagement of the 89 million fans of the genre. Retweeting posts from racial justice movements, in contrast, may feel like a way to do a more progressive form of participant ethnography that gives back to community activists by publicizing their work. These same forms of digital participation look very different for researchers studying far-right movements and hate groups. As Forberg discovered in his research, QAnon content typically includes scientific misinformation about vaccines or an overview of the type of government conspiracies about election theft that spurred the January 6th Insurrection at the Capitol Building in 2021. Liking a QAnon post on TikTok, Instagram, or Twitter may contribute, however marginally, to that poster's broader success on the platform, monetarily reward the poster, and vindicate the poster's beliefs. The risks of such participation are exacerbated by the possibility that the ethnographer has adopted an anonymous persona where they may be expected to engage in potentially harmful activities. In analog ethnography, IRBs would likely take issue with a researcher picketing for an anti-vaccination protest, even if this protest was seen by only a handful of people. To push the comparison further, for QAnon, the most carnal or embodied form of participation—developing conspiratorial interpretations and posting recruitment material online—is eerily similar to the analog 1950s doomsday cult recruiter, with the exception that this performance could happen almost completely in physical isolation. For digital ethnography, then, we must investigate more fully the ethical line around peripheral and embodied participation, such as retweeting an anti-vaccination video or liking an anti-vaccination post, that can spread misinformation at a large scale. While internet users may adopt anonymity or active disguises with impunity, we believe that researchers should hold themselves to a higher standard when it comes to making decisions about participation in digital ethnography. We should always ask ourselves what effect our disguise or persona could have in these virtual communities to avoid reifying the fallacy that what we do in the digital world is less real or impactful than what we do in the “real world.”

## 4. Conclusion

In this article, we pose the question, “What is ethnographic about digital ethnography?” Working through our cases and examples, we suggest that a digital ethnography should share the same set of techniques as what we call an analog ethnography—namely participant observation in a social world that occurs over an extended period of time. Whether an ethnographer is following a hard-to-reach digital community that has no analogous in-person setting, participating in a social world with people whose lives blur the distinctions between on- and offline, or studying neo-Luddites who do not use technology, she should aim for the direct contact or “exposure” with the social world and its people that is the defining feature of ethnographic research (Small and Calarco, 2022). This is not to say that digital settings lack unique forms of social interaction that are worthy of study. We think, for example, of the prevalence of anonymous death threats on social media that happen at a scale and volume we do not see in face-to-face interactions, or of the long life of poorly worded social media posts that can never really be erased from the internet. The rise of social media platforms, smartphone usage, and user-generated content also creates new forms of social artifacts that are ripe for sociological analysis every day. But we argue here, qualitative research done through digital platforms is not by default digital ethnography. We make this point, following Lamont and Swidler (2014), to encourage “methodological pluralism against methodological tribalism” (p. 154). As they remind us, “the selection of methodological approaches should depend on the questions being pursued— different methods shine under different lights, and generally have different limitations” (p. 154). Digital discourse and content analysis can tell us a great deal about temporality and historical change over time through rigorous analyses of social artifacts, whether this be Reddit forums, video game guilds, or Instagram posts. These methods do not allow for—nor do they need—the extended researcher co-presence we would expect from an ethnographic project to satisfy their epistemological stance. Even real-time observation of such digital spaces does not necessarily produce relational data between the ethnographer and participants.

We offered two questions that we think ethnographers must consider regardless of the modality of their research: First, is the ethnographer's identity as a researcher known to others in their field? Second, what is the ethnographer's role in the field? Decisions about how to present oneself and how to participate are necessarily informed by one another, as well as by ethical, epistemological, and contextual concerns. Juxtaposing Forberg's digital ethnography of QAnon and Schilt's analog ethnography of support groups, we have shown how digital ethnography raises familiar questions about research strategy at the same time that it provides new affordances for self-presentation and co-presence. What the researcher seeks to know, where she looks to answer this question, and how she understands the norms and expectations of this setting should shape her approach to self-presentation and degree of participation in digital and analog work. A decision about how to present yourself in the field can change over time, as Forberg's research shows. And, in some cases, deep cover is not possible or ethical, as Schilt's research shows. What unites both approaches in our view is a careful consideration of the epistemological stance of the research, the possible risk to the researcher depending on her approach to self-presentation and co-presence, and the possible harm to respondents, the broader discipline, and society at large that a study poses.

To us, ethnographers across modalities must be aware of the self-presentation norms of a particular setting. Anonymous lurking online may more closely align with some digital community norms (Ferguson, 2017). Yet, the internet is also becoming increasingly authenticated, with users posting under real names and providing searchable, personally identifiable information (Barratt and Maddox, 2016). The increase in reverse image searching and widespread availability of tools for doxxing also means almost anyone is “unmaskable” on the internet. The growth in government and personal security cameras also limits analog anonymity, as we saw in cases of the police using facial recognition software and crowdsourcing video footage to identify protesters during the social unrest around racial injustice in the summer of 2020 (Vincent, 2020). And, in any form of disguised or anonymous research, anonymity evaporates as soon as data is published online. While researchers may strategically create profiles online that provide access to communities and help build rapport with participants, we caution against developing fake personas when trying to understand real people. Instead, we suggest that participation should focus on building relationships with participants, learning about their practices through them, and participating in acts such as content creation or video game playing when it is appropriate and meaningful to do so—which in many cases will require that the ethnographer makes herself known to the participants as a researcher.

In building known relationships with participants, ethnographers should also be cognizant of the behavioral norms in their research setting and be reflexive about how their degree of participation—whether that is lurking, liking, or tweeting in a digital space or observing, nodding, or engaging in an analog space—can transform the group dynamics. Lurking online or observing a physical space anonymously over time can be a diagnostic tool that helps triangulate other forms of data (Duneier, 1999), or it can be the main source of data about a community. In many digital spaces, peripheral forms of participation such as liking, following, or viewing often are appropriate to the norms of the setting, and can produce valuable data about how digital systems work and respond—especially in algorithmically-mediated environments where any degree of presence necessarily incorporates users into the algorithm's all-consuming logic. However, in certain spaces, such as in deviant digital spaces, the ethnographer may be liable for supporting harmful content and vindicating harmful users, or, if using a disguised approach, be pushed to produce harmful content akin to 1950s graduate students encouraging new cult members to give up their worldly possessions. In our view, decisions about self-presentation and co-presence should not be made on the basis of what is possible in a setting but rather on the basis of what is both ethical and efficacious for gathering quality data.

We end this article with the acknowledgment that our distinctions between ethnographic research modalities may seem to some readers already outdated. We use “digital” and “analog” as heuristics but recognize the impossibility of a firm distinction in many social contexts. As the many works cited throughout this piece demonstrate, the use of the term “digital ethnography” does not foreclose mixed-methods techniques that work across digital and analog contexts, leverage big data, or study drastically different digital environments—as the ultimate goal is finding the method that will suit the research objectives. However, at this moment, in proposing digital ethnography vis-à-vis analog ethnography we hope to both elevate digital ethnography within sociology and to reiterate that present social contexts require intentional approaches to understanding the role of digital systems in the social world. We can envision a future in which the proliferation of digital technology has rendered such distinctions between modes of research entirely moot—perhaps in a universe where the proposed “metaverse” replaces analog spaces such as schools and office buildings. In this version of the future, all ethnographic research might be hybrid. But, as we write this in 2023, many of the institutions that produce and maintain the rampant structural inequality in our society, such as prisons, public housing, or financial institutions, remain difficult to study without physical co-presence. Further, the most vulnerable people in our country, including people who are incarcerated, elderly, unhoused, or living in deep poverty, have difficulty maintaining a digital presence through a smartphone, a social media account, or even email, making digital forms of research less possible or applicable to large parts of the social world. Our suggestion is that ethnographic research in sociology should fit the reality of the people's lives we are studying, adopting a single-mode or hybrid approach as appropriate to the context. Hybrid ethnography may indeed be the future, but we would do well to remember, paraphrasing science fiction writer William Gibson, that the future is never evenly distributed.^7^

## Author contributions

All authors listed have made a substantial, direct, and intellectual contribution to the work and approved it for publication.

## Footnotes

^1^While we see sociology in the U.S. as being slow to acknowledge how the internet was changing social interactions at a societal scale, we want to acknowledge here a few notable sociologists who saw the importance of studying online cultures long before this current wave of digital ethnography. T.L. Taylor published a qualitative study of online gaming culture in 2006 and was a co-editor of *Ethnography and Virtual Worlds* (Boellstorff et al., 2012). Dhiraj Murthy (2008) made early appeals to the need for more digital qualitative research in sociology, and Kozinet (2011) produced the first how-to book on internet ethnography in sociology.

^2^Our title question is a reference to Aspers and Corte's (2019) article, “What is qualitative about qualitative research?” Taking insight from Small (2021) critique of the article, we recognize the impossibility of providing a definitive answer. We offer instead some ways forward for a broader discussion.

^3^Much of the initial discussion of digital ethnography focused on “online,” “internet,” or “virtual” communities. This language necessarily persists in our discussion of this literature. We view these terms as specific subsets of digital ethnographic inquiry more generally.

^4^“Doxxing” is the release of someone's personal information, such as telephone number or home address, online. The term comes from 1990s hacker lingo for releasing classified or sensitive documents stolen from a computer.

^5^Here we can think about, for example, the rise and fall of Myspace, LiveJournal, and Tumblr in terms of users, or the attrition of young people from Facebook and Instagram in favor of TikTok. And, at the same time, a how-to for TikTok ethnography would be short-lived.

^6^For digital ethnography, we can also think about “audible” tools, as many networked social media sites allow for voice chat.

^7^As Kennedy (2012) notes, this quote may be apocryphal, but it sums up much of Gibson's philosophy in his writing.
